# Adolescent substance use: Findings from a state-wide pilot parent education program

**DOI:** 10.1186/s12889-022-12899-2

**Published:** 2022-03-21

**Authors:** Nayantara Nair, Alishia Elliott, Sarah Arnold, Andrew Flachs, Barbara Beaulieu, Kristine Marceau

**Affiliations:** 1 Department of Human Development & Family Studies, Purdue University, 1202 West State Street, West Lafayette, IN 47907 USA; 2 Department of Psychology, Valparaiso University, Valparaiso, USA; 3 Department of Public Health, Purdue University, West Lafayette, USA; 4 Department of Anthropology, Purdue University, West Lafayette, USA

**Keywords:** Adolescent substance use, Parent-education, Community programming, Pilot study, Focus group discussions, Qualitative analysis

## Abstract

**Background:**

Adolescent substance use has long been a top public health priority. In Indiana, concerning recent trends show high rates of youth alcohol consumption coupled with increasing use of opioids, synthetic marijuana, and over-the-counter drugs. Based on research indicating that parent-based prevention efforts may be a particularly effective way to target adolescent substance use, and in a direct effort to address Indiana’s 2017 Strategic Plan to Address Substance Use, we conducted an applied research study targeting parents’ knowledge regarding adolescent substance use in Indiana.

**Methods:**

This community-based applied research study included: (i) a needs assessment of Indiana Extension Educators’ concerns regarding adolescent substance use, (ii) creation and dissemination of an evidence-informed parent education program on adolescent substance use in collaboration with Purdue Extension (a key community stakeholder), and (iii) qualitative focus group discussions at the end of each program that assessed the challenges families face regarding adolescent substance use, the types of information and resources they wish they had, and the usefulness of our program.

**Results:**

The needs assessment revealed that Indiana communities would most benefit from education regarding ways to spot and monitor substance use in teens, and strategies to communicate with teens about substance use. Additionally, Extension Educators thought that existing resources to tackle substance use largely did not match the needs of Indiana communities. Qualitative analysis of the focus group discussions across 8 pilot programs revealed five important themes: (1) The need for current, evidence-informed information regarding adolescent substance use among parents and youth-involved professionals in Indiana, (2) Concern regarding Indiana adolescents’ ease of access to substances and lack of healthy recreational activities, (3) Communicating with teens about substance use is crucial but difficult to implement, (4) Indiana communities’ need to prioritize funding for evidence-informed prevention programming, and (5) The need for community-based parent and caregiver support groups.

**Conclusions:**

Overall, the program was well-received and participants indicated that there was a strong need for this programming in their communities, but suggested collaborating with schools or similar local community stakeholders to increase attendance. Findings from this pilot study can inform future community-based adolescent substance use prevention efforts state-wide.

## Background

Adolescent substance use remains a crucial public health and policy priority, as it has been linked to several long-term adverse outcomes including continued substance use and dependence, mental health concerns, and other psychosocial adjustment concerns in adulthood [1]. Nation-wide U.S. data on adolescent substance use indicate that alcohol remains the most widely used substance among teens, and although rates of vaping have finally leveled and reversed after years of continued increases, marijuana use and use of other illicit drugs by 8th, 10th, and 12th graders has remained consistent [2]. Additionally, although several forms of tobacco use have declined, cigarette use remained stable in 2020 after a long period of decline [2]. In the state of Indiana specifically, rates of adolescent substance use are broadly equivalent to national trends. That is, rates of vaping across the state significantly declined in 2020 compared to 2018, as did past-month alcohol use for students in grades 8-11. Indiana youth’s marijuana use remained consistent across grades 6, 7, and 8 but decreased for students in 9th and 11th grades. Contrary to national patterns, cigarette use in Indiana has continued to decline [3, 4]. Of concern, however, are the steady increases in the use of synthetic marijuana among 6th -11th graders, and methamphetamine among 7th and 9th graders. Additionally, Indiana continues to see high rates of over the counter (OTC) drug usage, particularly cough syrup, among 7th -12th graders [3]. Given the widespread nature of the problem in addition to its well-established long-term consequences, substance use has been consistently highlighted as a top public health priority both nationally [5] as well as specifically in Indiana [6, 7].

### Rationale for proposed program

Well-established evidence indicates that adolescents are particularly vulnerable to the initiation of substance use and progression to problematic use [8–10], and that adolescence is therefore a particularly critical “at-risk” period for the development of substance use concerns. To target this increased developmental risk of substance use, researchers and public health experts have increasingly called for evidence-based and evidence-informed preventative interventions that can delay early use of substances and halt the progression from initial use to problematic use [5, 11, 12]. Further, literature indicates that universal school-based prevention interventions may be less effective in curbing adolescents’ use of alcohol and tobacco (particularly prevalent substances in Indiana) compared to that of illicit drugs [13–15]. On the other hand, parent-based efforts have been found to be particularly useful in preventing alcohol, tobacco, and cannabis use among 10 to 18-year-olds [16].

In Indiana’s 2017 strategic plan to address substance use [17], Governor Holcomb’s specific proposals included to: 1) *“Identify and support the implementation of age-appropriate evidence-based addictive substance use and misuse prevention programs for children and youth. Encourage school-based programs that support positive peer relationships and social competence and evidence-based family strengthening programs”*, and 2) *“Encourage and support community-based coalitions aimed at prevention, treatment, and recovery. Encourage significant involvement of community-based organizations, Purdue Extension, chambers of commerce and other organizations from the public, for-profit, and not-for-profit sectors.”* In a direct effort to address these recommendations, we developed an evidence-informed program to increase parents’ knowledge and awareness of strategies that have been successful for preventing or reducing adolescent substance use. As Indiana’s land-grant university, one of Purdue’s main missions along with research and teaching is extension: i.e., direct engagement with community stakeholders in efforts to improve Indiana residents’ livelihoods through evidence-based services and resources. Purdue Extension, present in all 92 Indiana counties, is therefore tasked with the dissemination of current, evidence-informed programming across the state to tackle Indiana communities’ most pressing needs. Collaborating with Purdue Extension was therefore a crucial aspect in the design, development, and dissemination of this program.

### Development and design of the program

The overall goals of our ‘Adolescent Substance Use: Parent Education through Extension’ program were to 1) better understand adolescent substance use, specifically with regard to the needs of Indiana residents as indicated by a key service provider: Purdue Extension Educators and 2) to provide training for Purdue Extension Educators in order for them to disseminate research-based information to underserved Indiana communities about what families can do to prevent adolescent substance use. Importantly, by taking into account the target population’s needs as well as collaborating with a key community stakeholder, our study borrowed key design and implementation principles from Community-Based Participatory Research (CBPR). CBPR involves a collaborative process in which researchers, community members, and stakeholders are equitably involved in the research process, with the aim of combining knowledge and action for social change to improve community health and well-being [18–20]. The program therefore consisted of three phases that incorporate elements of CBPR to achieve its goals:

I. *Information Gathering/Needs Assessment *In line with the core tenets of CBPR, our first aim was to conduct a needs assessment to understand the specific adolescent substance use-related concerns that Indiana residents face. Therefore, in the first phase of the project we gathered information from Purdue Extension Educators through Qualtrics surveys regarding adolescent substance use concerns both in their own communities as well as in Indiana communities in general. Specifically, we asked: a) whether they think adolescent substance use is a problem, b) which substances are of particular concern, c) whether communities would benefit from educating parents and/or providing them with resources about adolescent substance use, d) what specific topics parents would benefit from learning about, and e) whether there are previously established prevention programs that adequately meet the needs of the community. We then created a parent-education program based on seminal and current evidence pertaining to the topics that Extension Educators were particularly concerned about regarding adolescent substance use.
II. *Education through Extension *In the second phase, we invited Purdue Extension Educators to come to one of two centralized training locations where we presented our evidence-informed parent education program in full-day training sessions that included information on current rates of substance use nationally and in the state, current substances of concern and substance slang, key predictors of substance use (including adolescent characteristics, adolescents’ close relationships, and availability of and access to substances), and corresponding real-life tips that can be implemented by parents to prevent substance use in their teens. While presenting this information, we trained educators to deliver this program to families in their own counties. After the training programs, interested Extension Educators then conducted the program for parents in their own Indiana counties.
III. *Qualitative Program Evaluation* Two research assistants (RAs) accompanied the Extension Educator for each of the eight parent-education programs conducted across the state. After each program, the RAs conducted a town-hall style focus group discussion with the participants, asking them a series of open-ended questions designed to probe the kind of challenges families face regarding substance use, the types of information or resources they wish they had, and how useful they found our program. Consistent with a CBPR approach, our aim was to engage community members in every phase of the study in order to include their experiences and perspectives on the program content and style of delivery prior to expansion.

## Method

This applied research study was conducted by an interdisciplinary team of researchers and field-based personnel to address adolescent substance use in Indiana through a multidisciplinary approach that included quantitative, qualitative, and community-based applied research methods.

### Research design

The current study was based on an inclusive, community-based applied research project, with elements of its design and implementation borrowed from CBPR principles. Specifically, the study included an assessment to identify the target population’s needs regarding substance use and collaborated with a key community stakeholder (Purdue Extension) in the design and implementation of the parent-education program. Emerging systematic reviews of the literature and meta-analytic evidence indicate that interventions, programs, and policies that emerge from projects that involve community input may have improved internal and external validity [21] compared to those that are based purely on investigator-led research designs. With this guiding framework in mind, we collaborated with Purdue’s Extension network and Indiana community residents to: a) gauge community priorities with regard to adolescent substance use, b) disseminate evidence-informed information to Indiana parents, and c) evaluate Indiana residents’ adolescent substance use concerns, the ability of existing resources to match communities’ needs, and our program’s usefulness and feasibility to scale up to the state level.

### Participants and procedures

The study protocol for Phase I and III (the phases involving research with human subjects) was approved by the Purdue University IRB, protocol #1806020709.

I. *Information Gathering* This phase of the project included a survey of Health and Human Sciences and 4-H Extension Educators across the state, as these are the educators that regularly engage with families on topics of family life. Extension Educators were contacted directly via email with a link to the educator survey. The brief (3-5 min) Qualtrics survey obtained their opinions on the scope of the problem of adolescent substance use in their community and Indiana as a whole via both closed- and open-ended questions. Informed consent was provided via Qualtrics at the start of the survey. Educators were not required to provide identifying information, but they had the option of providing contact information if they were interested in participating in the second phase of the project. Out of approximately 150 educators on the listserv at the time, 87 opened the link and 71 completed the survey.

II. *Education through Extension.* Of the Extension Educators who completed our information gathering survey, 27 indicated that they were interested in participating in Phase II of the study. We invited these educators as well as educators from all other HHS and 4-H Extension offices from across the state to participate in one of two full-day sessions (in the Northern and Southern parts of IN) that focused on training educators to deliver our evidence-informed adolescent substance use parent-education program. In total, educators from 15 different counties attended our training sessions. Nine of these educators then went on to deliver the program in their own counties across the state.
III. *Qualitative Program Evaluation.* Participants of the third phase were attendees of the programs that were disseminated by Extension Educators in Phase II. Two RAs attended 8 of the 9 programs that were disseminated across the state and conducted a town-hall style focus group discussion at the end of each program using five open-ended questions developed by the research team to assess participants’ concerns regarding adolescent substance use, the information and resources they indicated they needed, and the usefulness of our program (See Table 1). Focus groups are a well-validated technique to obtain information during program development and evaluation [22, 23], have been established as an effective method of data collection with parent populations specifically [24], and are considered to be particularly useful in the context of sensitive topics when there is an opportunity to solve a pressing problem [25].

**Table 1:** Educator needs assessment and focus group discussion questions

No.	Educator Survey Questions (Qualtrics)	Focus Group Discussion Questions
**1**	Do you think there is a problem with adolescent substance use in your community/IN communities in general?	We would like to hear a little more about why you came here today. What were you hoping to get out of coming today?
**2**	Which substances do adolescents in your community/other IN communities use?	Could you tell us about the adolescent substance use issues you see in your community?
**3**	Do you think your community would benefit from educating parents and/or providing them resources about adolescent substance use?	Is there something you would take from today’s workshop that you can use?
**4**	If yes, how much do you think your community would benefit from knowledge regarding: rates of use, how to spot and monitor use, communicating with teens about substance use, substances that are specifically an issue, etc.	What tips do you have to improve this program?
**5**	Are there other programs already in place? Do they meet the community’s needs?	Any other feedback?

All program attendees were invited to participate, but participation in the focus group discussion was not required for attendance at the program; it was voluntary and introduced only after the program was complete. Attendees who chose to participate in the focus group discussions (see Table 2 for demographics) tended to be youth-involved professionals and likely represent the most enthusiastic and engaged members of the target population. Specifically, they were the most invested in adolescent substance use concerns within the community and included teachers, foster caregivers, youth program specialists, and others with intimate professional connections to at-risk youth. Attendees were asked to sign an informed consent form, and discussions were audio-recorded with the consent of the participants. No identifying information was included in the discussions, but while one RA moderated the focus group discussion, another took field notes regarding observable demographic details of the participants as well as participants’ non-verbal behavior during the discussions [22, 26]. The number of attendees who participated in the focus group discussions varied from county to county, ranging from 2 to 12 attendees across programs.

**Table 2:** Description of programs and participants across eight indiana counties

No.	IN County	County: Urban vs. Rural	Number of Participants	Participants: Parents, Professionals, or Both?
1	Cass	Rural/Mixed	3 (100% F)	3 both
2	Delaware	Urban	7 (71% F)	5 professionals, 2 both
3	Howard	Rural/Mixed	3 (67% F)	1 professional, 2 both
4	Marshall	Rural/Mixed	12 (92% F)	8 parents, 3 professionals, 1 both
5	Spencer	Rural	3 (67% F)	2 professional, 1 both
6	St. Joseph	Urban	7 (57% F)	2 parents, 1 professional, 4 both
7	Vanderburgh	Urban	10 (70% F)	3 parents, 3 professionals, 4 both
8	Wabash	Rural/Mixed	3 (67% M)	2 parents, 1 professional

All programs were conducted in 2019. ‘*M*’ Male; ‘*F*’ Female. Indiana County data received from https://pcrd.purdue.edu/ruralindianastats/geographic-classifications.php#second

### Qualitative analysis

Focus group discussion recordings from Phase III were transcribed by undergraduate RAs, and transcripts were double-checked by a third RA for accuracy. These transcripts were then coded using Saldaña’s [27] qualitative coding methods and guided by a codebook approach to thematic analysis [28, 29]. The development of the codebook and the coding process were collaborative and occurred in several stages. First, the two RAs who were present at each of the focus group discussions read the transcripts, generated an initial set of codes from that data, and organized those codes into meaningful groups according to deductive themes based on the focus group discussion questions. This hierarchical coding framework served as the initial codebook [30] for analysis. Second, the two RAs pilot-tested the codebook using a subset of the transcripts as well as field notes from the focus group discussions, and identified additional emergent codes and themes using an inductive, grounded-theory analysis [27]. The codebook was then revised and finalized before formal coding began, and included code descriptions and definitions, inclusion and exclusion criteria, and example quotes from the data. In the third stage, each transcript was double-coded independently by two RAs across the deductive and inductive themes identified in the codebook, and then consensus coded by the RAs who conducted the focus group discussions. Using QDA Miner, the RAs identified five themes that crosscut the eight focus group discussions using inductive coding techniques.

To ensure data trustworthiness and credibility throughout the coding process, a number of steps were taken: i) the RAs involved in the final coding and analysis stages were also present at the focus group discussions and communicated regularly with members of the research team [24]; ii) all codes and themes were checked through peer review with a member of the research team who has extensive qualitative research experience [31], and iii) method triangulation (using multiple sources of data including transcripts, field notes, and observations), and researcher triangulation (participation of two or more researchers in the analysis process) were used throughout the coding process [32].

## Results

### Needs assessment survey

As reported in the Qualtrics survey, most Extension Educators (94%) responded that adolescent substance use “is a problem in my community”. Alcohol was the top substance used by adolescents, rated by 79% of educators, with tobacco (69%), marijuana (68%), opiates (48%) and stimulants (42%) also relatively frequently endorsed. Educators also frequently endorsed substance use “in other communities in Indiana”, with these same five substances rated highly (>70%), and additionally 50-60% of educators endorsed that adolescents in other communities used inhalants and sedatives.

The vast majority (95%) of educators thought that their community would benefit from educating parents and/or providing resources about adolescent substance use. Of that 95%, the top-ranked priorities for parents’ education included how to spot and monitor adolescent substance use (49% endorsed as the most important) and communication with their adolescent about substance use (25% endorsed as the most important). Educators less frequently endorsed substances adolescents use to alter mood, self-medicate, or get high (10%), data on rates of adolescent substance use (10%), or substances specifically an issue in their own community (4%) as the most important topic. Other suggestions (e.g., from text entry) included information on the effects of vaping and information on juuling, the risks of providing minors alcohol at your home, safe disposal of medications, mental health generally, stigma related to drugs, and community resources and initiatives to empower youth.

 Just under half (45%) of educators said there are programs for parents on adolescent substance use already in place in their community, but of that 45%, only 58% of those programs matched the community’s needs. Outside of knowledge-building programs for parents, most educators thought that after-school programs (88%), opportunities for adolescent employment (86%), support groups (83%), and rehab facilities (67%) would match their needs. Other suggestions of resources that would fit their community’s needs included 1) online/tech-based information (i.e., social media clips, online materials/options, outreach via technology, information on communication without stigma, actual stories of families affected), 2) services for adolescents with substance use problems (i.e., court-ordered classes, navigation services, 24-hr hotlines), and 3) positive youth development programming (i.e., empowering programming for kids, positive youth development, healthy recreational activities). These educator survey results informed the development of the parent program.

### Program reach

In total, Extension Educators disseminated the adolescent substance use parent-education program in eight counties across Indiana from June-October of 2019. Most of the counties in which the program was disseminated can be classified as ‘rural-mixed’ (i.e., rural with some larger towns; [33]). The number of participants that attended each program ranged from 2 to 12, and participants were mostly women (see Table 2).

Although we initially intended the program to be for parents of teenagers, we found that it drew a diverse range of attendees who had either personal or professional ties to youth in some capacity. That is, in addition to parents, attendees also included teachers, youth workers, CASA (Court Appointed Special Advocates) volunteers, foster-care caregivers, as well as grandparents and other extended family members who had teenagers in their immediate circle.

### Qualitative focus group interviews

#### Deductive themes

**Fig. 1:**
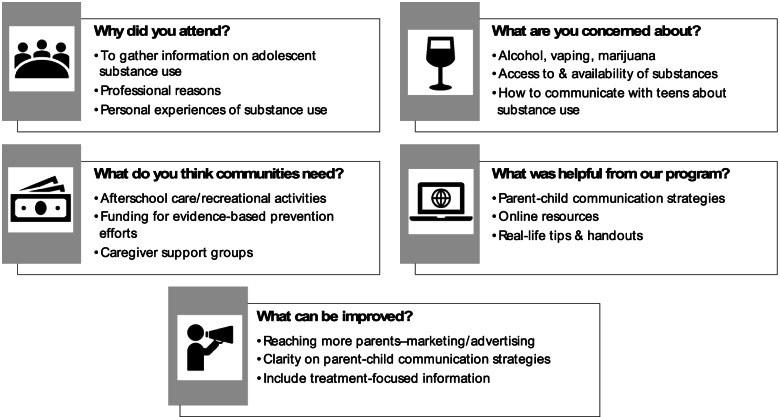
Deductive themes drawn from qualitative focus groups

**Fig. 2:**
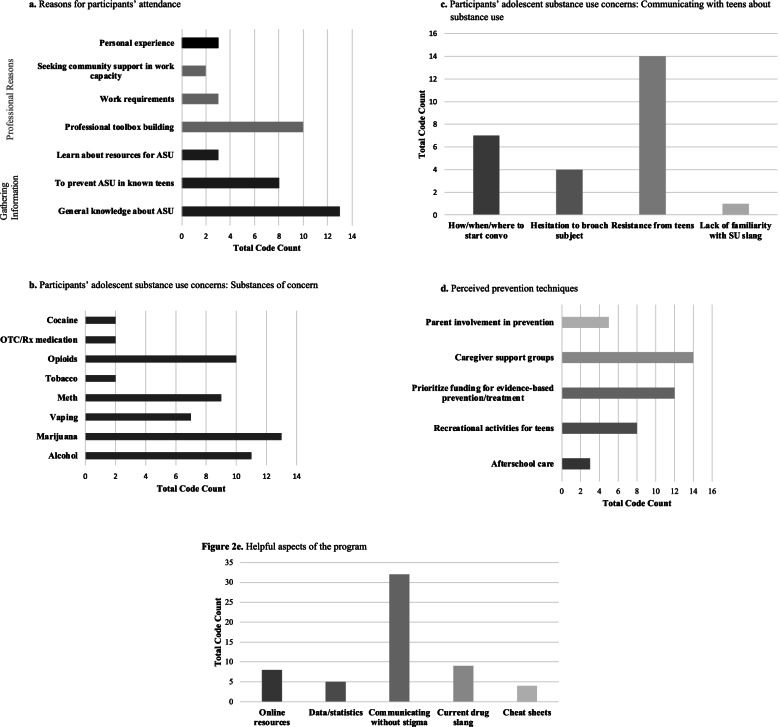
**a** Reasons for participants’ attendance. **b** Participants’ adolescent substance use concerns: Substances of concern. **c **Participants’ adolescent substance use concerns: Communicating with teens about substance use. **d **Perceived prevention techniques. **e **Helpful aspects of the program

The questions asked during the qualitative interviews along with summaries of the key thematic responses are presented in Fig. 1 (also see Fig. 2 for a breakdown of code frequencies for each theme across programs). In general, participants expressed positive views with regard to the program content and mentioned finding the information, activities, and handouts both useful and enjoyable. As an example, one participant stated “And it was an eye opener, it was great and the overheads and the things you passed out was just real informative and it was… I enjoyed it. And I think more parents should be aware of this.” They appreciated that the program provided up-to-date statistics and real-life tips for parents based on current evidence and was presented in a way that was applicable to a wide range of audiences (i.e., parents as well as anyone working with youth in a professional capacity). As discussed in the inductive themes in the next section, participants especially appreciated that the program tackled a key substance use concern: communicating with teens about substance use without stigma. For instance, one participant reported: “I feel like there was [sic] a lot of positive things I’ve took away [sic] from this evening… but I think, overall, just the dynamics of the parent-youth relationship was very good.”

Attendees also discussed aspects of the program that could be improved on, particularly, how to expand the program’s reach and encourage more parents to attend. In 7 of the 8 focus group discussions conducted (i.e., in all except Marshall County), the majority of program attendees were youth-involved professionals rather than parents. This included educators, foster-caregivers, social workers, etc., although many of these attendees were also parents. Attendees therefore discussed the need to strategize ways to incentivize the program or make it more convenient for parents to attend. For example, participants suggested that the program would lend itself well to a virtual format with online resources and e-handouts provided. They also suggested improving the marketing and advertising of the program through schools, churches, and other local community stakeholders to reach the program. Content-based suggestions, although fewer in number, mainly centered on bolstering existing strengths of the program or components that were considered particularly useful: for example, participants suggested including real-life examples of parents and youth modeling how to communicate about substance use without stigma:

I would like to see a live—like to bring in a child and adult next time give us example dialogue on different things. I know you touched on that earlier, it’s actually a good idea to have them in front of the group, that might be helpful.

Additionally, participants’ suggested content additions included intervention tips for when youth are already using and/or facing substance use concerns, more information about the biological/genetic aspects of addiction, and showing physical samples of the substances adolescents commonly use.

#### Inductive themes

In addition to the deductive themes that arose in response to explicit research questions, the research team also identified a number of inductive themes from a grounded-theory analysis of the focus group transcripts. These inductive themes centered around five key ideas. From the perspectives of participants as not only parents and family members but also service providers who work with youth, discussions broadly focused on (a) participants’ need for current research-based information on the problem of adolescent substance use, (b) participants’ concerns regarding specific substances of concern as well as adolescents’ underage access to substances, (c) communication as a key concern with regard to adolescent substance use, (d) Indiana communities’ need for prioritized funding for research-based intervention and prevention efforts, and (e) mixed evidence regarding the need for parent/caregiver support groups.

##### Theme 1: The Need for Current, Research-Based Information Regarding Adolescent Substance Use among Parents and Youth-involved Professionals

Many participants indicated that they attended the program to access up-to-date and research-based information on substance use in teenagers. This includes information on substances and substance-related slang that teens may use, data regarding rates of substance use, and well-established parenting tips for preventing substance use in their teens. For instance, one participant reported: “Well for me it’s, a lot of it for me is data. I need to know what’s happening today, and what’s recent data showing?” Another stated,

I’m the parent of a[n] 18-year-old female and a 16-year-old male, and I also work with youth from the ages 10 to 17. I wanted to know what current information was available, and if my parenting is on track maybe? Or [if] research indicated that it makes sense.

Similarly, a participant explained the lack of knowledge she felt she had about substance use and the need for as much information as possible to be able to deal with it with her children:

I think we need to get more classes like this one because you can get more information, like for me I have three kids. And one is a teenager…and we don’t know anything about it, like especially for me like a mom we don’t know anything about it. All that we can learn right now is you know, the different ways, the different names, so that’s why we come [sic] here to learn about it.

The need for evidence-informed information was particularly highlighted by attendees who worked with teens in a professional capacity, such as educators, foster-caregivers, CASA volunteers etc. For instance, one attendee stated “I am a school counselor and so, I wanted to get more information that I thought would be helpful to share with parents or staff…’Cause that’s the hardest part is keeping updated on everything.” Another noted, “I also work with children and families and we see a wide array of family issues and how that impacts children, so…just more research, more interventions that we can recommend, and use with families and kids.” Other attendees who were involved with youth in a professional capacity specifically indicated the need for more current evidence-informed information to bolster their own work-related skills:

Yeah, for me, I’m, being a prevention specialist and working a lot with youth, any opportunity I have to enhance those skills, learn a bit more about new evidence-informed programs and ideas and theories, and just be in a room with people who are sharing their experiences, and what they’re trying to get out of it as well, so that’s kind of [what] my intentions were here today.

##### Theme 2: Concern Regarding Indiana Adolescents’ Ease of Access to Substances and Lack of Healthy Recreational Activities

Attendees across all eight programs noted their concerns regarding the easy availability of and access to substances for youth in Indiana, particularly with regard to alcohol, tobacco, and marijuana. Specifically, attendees mentioned informal sources of access to legal substances through parents, siblings, and peers, with one attendee stating “These parents are allowing their kids to use it [alcohol]. They have older siblings that’ll just give it to them because it could be seen as a rite of passage. It could be seen as not that big of a deal.” Attendees also mentioned adolescents obtaining substances through formal sources, such as this participant who said: “I think someone mentioned earlier, you know, gas stations are, you know, you can kinda find like a couple of gas stations where we can either get like, like tobacco underage…”. Additionally, attendees were particularly concerned about whether the legalization of marijuana would increase adolescents’ access to and availability of that and other drugs. For instance, one attendee stated:

Well, that’s a problem too, that, you know, the law will change. Marijuana will be legal in this country, just in a moment, but the other drugs, you know we’re not, I…I wouldn’t preach what some countries do in Europe, they just legalize all drugs, but something has to be done to stop the availability, and the use of these hard drugs that kill.

Participants were also concerned that the discussion surrounding marijuana legalization has changed teens’ perceptions of the drug. As one participant reported, “I was gonna say marijuana ‘cause, especially now, like my daughter justifies it all, ‘like oh in the state of Colorado, it’s legal, and it’s medical use’, and you know, just all that glorification…where it’s now become the norm.” This concern regarding adolescents’ access to substances in Indiana communities was also coupled with attendees’ worries about the lack of healthy recreational activities that teens, especially in rural parts of Indiana, had access to. One participant stated:

I think in my opinion since alcohol is easily accessible, because parents who drink and as a general statement- in the Midwest in a small town, if kids don’t find positive activities, drinking is easy to get ahold of or access and plus peers brag about having access or saying they have done something. Everyone wants to feel accepted.

Similarly, another participant reported: “‘Cause there is *[sic]* school functions, but, outside of those teams or extracurriculars, in rural communities, in southern Indiana, there’s not positive activities for them to do, so that they’re turned to alternatives.”

##### Theme 3. Communicating with Teens about Substance Use is Crucial—But Difficult to Implement

In all eight programs, attendees mentioned finding it difficult to talk to their teens about substance use. Specifically, parents mentioned their teens not wanting to open up to them about the subject, or being resistant to their efforts to tell them about the risks associated with substance use. For instance, one participant stated: “As a—as I got two of mine going to college so, as a mom…just that, the knowledge of how to bring those things up again even though they—the child’s like ‘I know!’” Attendees also mentioned the difficulties in just beginning a conversation about this subject with their teens, such as this parent who said: “I communicate with my daughter but to really talk about some of the tough subjects…I think it’s hard to get started sometimes”. Or, participants described difficulties in sustaining their teenagers’ attention during such a conversation:

That’s—that probably is the main problem. Well not just with substance abuse, but with behavior in general with kids anymore. How do—how do you even get their attention anymore, let alone hold it then try to tell them that, you know, the power’s in you to do good or evil and you need to work hard to succeed?

This observation was particularly highlighted among attendees who experienced trouble broaching the subject with teenagers who were already engaging in substance use. For instance, one attendee shared the following:

Um, but also the subject too, it’s something where I’m also an adoptive parent through the foster care system and have an oldest daughter that’s gotten in trouble with stuff, with substances, and she also has wrath, and so like the relationship piece…that’s why the role playing was painful, because my daughter would not respond—it’s ‘I hate you’”.

Participants also discussed their struggles to find “the right way” to have a conversation about substances with adolescents, especially since what works with one teenager might not work with another. As this parent described:

I think another thing that’s been sort of an issue, that, I mean that if I went to D.A.R.E, and there’s just an—I don’t think there yet is a right way to talk to kids about this. If you can sit here and tell kids ‘Just say no’, they’re not—it’s not gonna work. You can sit here and tell kids ‘Hey, it’s not cool to do this!’ they’re—it’s not gonna sit with them. I definitely don’t have the answer, but I don’t think there is just a—I don’t think there’s been a right conversation yet to have with kids, and every kid’s different.

Similarly, another parent stated: “That, and every kid and family is different…so how you approach it [the topic of substance use] is gonna be based off the kid’s personality, the family’s personality and their dynamic.” Complementing participants’ feelings regarding the difficulties they experienced in communicating with their teenagers about substance use, was the finding that the “Communicating without Stigma” section of the program appeared to be the most helpful aspect across all eight counties. For example, one parent appreciated the opportunity “to have more communication and also open questions, you know, because sometimes they [adolescents] say ‘no, just no’, so we need to learn how to start the question and with an open question without fighting”. Other participants found that the tips offered in this section of the program were particularly useful in learning how to engage teenagers in the conversation without having them shut down: “I think communicating without stigma was really good, on how to, you know, not be judgmental to, to the kids who are having the issues because that just makes them shut down.” Similarly, another learned that:

You need to not—you need to use I-messages and that can be really hard when you’re at a point where you’re not happy with what their choice was, but to use an I-message and to not um completely push them away.

##### Theme 4. Need to Prioritize Funding for Evidence-Based Adolescent Substance Use Prevention and Intervention

Another major theme that was revealed across programs was Indiana communities’ need for increased funding for evidence-based and -informed adolescent substance use prevention/intervention programs and parent support groups. Participants noted the decline in funding for substance use treatment facilities and programs over the years, with one stating:

And you know and for those who don’t ultimately—aren’t able to avoid the problems or abuse, you got to have some way to treat them. And you know that’s all I hear, there’s not enough treatment, there’s not enough facilities, there’s not enough, you know, rehabilitation services…there’s just* no funding for that kind of stuff unfortunately*. You know if there’s some way to get more programs available and communities that do um get into it.

They also noted a decrease in funding over the years for previously established prevention and intervention programming in Indiana communities. For instance, as one participant noted: “I know like through our work, we used to have like an after-school program that we would, like, help fund and take care of but it’s just, you know, a lot of the funding has disappeared over the years.” Similarly, another participant talked about the need to prioritize after-school programming at the state level in order to prevent adolescent substance use earlier on:

We failed academically, they ran out of money and now the state of Indiana is running it, but they’re not providing any more money, they wanted everybody to go out and get in their pocket and fund public education, but some of these neighborhoods that do them—after-school programs—you see progress, not only in academics, but social, socialization too, and it works, and they should, you know, if the state or the university’s going to make this a model, they still need to start earlier with the kids, and they need to do something, especially if they can identify these kids that don’t have any place to go after school….

##### Theme 5. Need for Parent/caregiver Support Groups: Mixed Evidence

Participants’ concern for the lack of funding for evidence-based prevention and intervention programming for teenagers also extended to the need for increased parent-based and educator-based programming in Indiana communities to effectively tackle adolescent substance use. This included mentions of the need for more parent-education programs like this one, as well as for support groups for parents and caregivers. Specifically, participants noted that building a sense of connection among parents, and providing parents/caregivers with a structured session to discuss common teen-related concerns they face, would be particularly helpful in addressing adolescent substance use in the state. For example, as one participant said, “I just keep thinking about the parents supporting each other and having the same philosophies about things, the network, the parents. I just think that’s real important. I think just, being comfortable talking to other parents about things going on and um, I don’t know….”. Similarly, another parent stated: “so I think when you have connections to other moms, other parents that ‘hey what works for you’, I think that would be important…on how to get them started and to have effective conversations”. Across the programs, participants mentioned that facilitating a sense of community among parents through structured and evidence-informed programming would play an important role in preventing adolescent substance use, such as this participant who stated:

I think the biggest thing when it comes to parents around this area is—it’s more so when it comes to substance abuse, is it’s seen as, just something that’s not that big of a deal, and like you said before, parents don’t normally talk to each other. So, I think there is that, that aspect of like, we need parents need to try to help other parents try to deter it.

However, we found that there were differences between counties with regard to the availability of programming and resources and whether or not they met the needs of Indiana’s youth-involved populations (e.g., teachers, grandparents, foster-care workers, etc.). Whereas some participants pointed to a mismatch between communities’ needs and existing resources:

And resources are very limited. There’s this huge waiting list to get help. So even the parent teacher support group—you know, we have one, and I made a note to-that’s something that would be good for them to do maybe. For them to reach out more to parents, talking about information, even continuing to educate the ‘kid leaders’ [quotes added] within their peer group. You know, kids who aren’t using—continuing to teach them how to influence their peers.

Others spoke of community stakeholders increasing the availability of support-based programming to match the needs of the community:

The other thing that has really blossomed, that I can say that our community is doing really resilient at, is the churches are jumping on board and providing support groups. So I think about the grandparent support group that has been created with movie nights and dinner fed to the family and then separation of you know the parents and the adults and the grandparents so that they can be ministered to at separate levels. But there’s also three different churches in our community that have started Celebrate Recovery.

Some participants noted that the problem is not the lack of available programming for parents and caregivers, but rather that attendance at these events tended to be so low that they could not continue to be offered:

We were actually just talking about this earlier, because we used to have a[n] adolescent substance use group that was ran weekly and it’s been years, five, what, five or more years since we’ve been able to keep that going and the problem isn’t going away, but the attendance and consistency of, pretty much families, enforcing that they’re coming to treatment, was not there, so we couldn’t even keep the group going.

The focus group discussions in this study revealed several notable findings regarding Indiana resident’s needs and concerns surrounding adolescent substance use. First, Indiana residents, for both personal and professional reasons, value access to more research-based information regarding the types of substances teenagers currently use, the rates of use among teens in the state, as well as the factors that may increase the risk of adolescent substance use. Second, a major concern among Indiana parents and youth-involved professionals centers on teens’ ease of access to substances, through both formal (e.g., stores) and informal (e.g., friends and family) avenues, particularly in the more rural counties that offer limited recreational activities for teenagers after school hours. Another key finding is that parents would especially benefit from learning easily applicable, practical strategies on how to communicate with teenagers about substance use. This was a topic that was consistently brought up both as a need, and an aspect of the program that was especially valuable in its potential as a prevention strategy. Parents commonly seem to face resistance from teens on discussing substance use issues, and would particularly value guidance on how, when, and where to start a conversation on the subject. Finally, the data indicate that there is a clear need to prioritize funding for adolescent substance use prevention and intervention resources, as well as parent/caregiver support groups, at the state-level. However, there also seems to be consensus (particularly among Extension Educators and other community programming professionals) that the residents who need these resources the most tend to be the ones who do not make use of them when offered. Therefore, prevention strategies may need to include not only the expansion of services across the state, but also enhanced advertisement and recruitment strategies.

## Discussion

This community-based applied research study aimed to create, disseminate, and appraise an evidence-informed parent education program to address adolescent substance use in Indiana. Importantly, we targeted one of the state’s top public health priorities, and in line with Indiana policy recommendations and the University mission, partnered with Purdue Extension (a key local community stakeholder) to help inform and disseminate our pilot program across the state. Overall, this program was well-received, with participants indicating that they learned valuable information such as current statistics, resources, and real-life tips for dealing with adolescent substance use in their homes and communities.

Findings from our pilot programs demonstrate that there is a strong need for evidence-informed programming tackling adolescent substance use in Indiana communities, not only for parents but for other youth-involved populations such as teachers, extended family caregivers, foster-care workers, etc. Specifically, we found that across all programs, Indiana parents, caregivers, and youth-involved professionals found our program particularly useful for: (i) access to up-to-date research and statistics regarding adolescent substance use prevalence, (ii) evidence-informed information on the risk and protective factors of adolescent substance use, and (iii) evidence-informed tips and strategies for ways in which parents can communicate with their teenagers about substance use (this was overwhelmingly endorsed by almost all participants across the eight programs). Although this last finding regarding the importance of parent-adolescent communication was particularly strongly endorsed among our study participants, there is mixed quantitative evidence supporting the association between parent communication and adolescent substance use. On one hand, a systematic review of parent-based intervention and prevention efforts targeting adolescent substance use found that successful programs were characterized by a central focus on improving parent-child communication, strategies for parents to implement boundaries, and improving parent monitoring of children’s activities [16], components that were incorporated into our program. However, meta-analytic results suggest that there is weak and/or inconclusive evidence of associations between parents’ general as well as alcohol-specific communication patterns and adolescent substance use [34]. Overall, researchers indicate that to better evaluate the effects of parent-based programming on adolescent substance use, more research that includes larger samples, control groups that do not receive the intervention, and longer follow-up periods is required [35].

With regard to Indiana communities’ needs surrounding adolescent substance use, our findings build on existing literature regarding state-specific adolescent substance use risk factors, as well as broader national recommendations for prevention. A report by the Center for Health Policy at Indiana University conveyed that community conditions that may exacerbate the risk of youth substance use in Indiana include a) the availability of alcohol and other drugs, b) community norms and laws favorable towards substance use and other risky behavior, c) low neighborhood attachment and community disorganization, and d) limited prevention and recovery resources [36]. Qualitative themes that emerged from our focus group discussion showed that participants were similarly worried about the ease of access to and availability of substances in the community, the legalization and consequent change of attitude towards marijuana, vaping, and other drug use, and the lower rates of community and parental involvement in prevention efforts as well as the limited availability of evidence-informed prevention and rehabilitation resources. Additionally, our findings are in line with national data suggesting that funding for community-based prevention and intervention efforts is a key concern at both the state and federal levels. Specifically, recommendations from national policy reports indicate that to effectively reduce adolescent substance use, funding is needed to a) inform and support parents at the community level, b) provide more school-based extracurricular opportunities for adolescents, and c) support the replication of school- and community-based prevention programs [37]. These needs are emphasized in our qualitative data, with participants specifically noting the lack of available funds in their community for prevention programming (particularly programming that is school-based and geared towards parents) and the limited recreational activities that Indiana adolescents have access to, especially in more rural counties.

Apart from confirming findings highlighted previously in the literature regarding adolescent substance use prevention needs, our pilot study revealed that parents in Indiana communities especially appreciated information regarding current national and state statistics teen substance use, the types of substances most commonly used by teens, and the language or slang teens use when referring to substances and different methods of use. Since a majority of the programs were disseminated in rural or rural/mixed counties (see Table 2), this could be an indication of reduced access to information and resources surrounding substance use in these Indiana areas particularly, as well as the reluctance to discuss and engage with the topic of substance use due to its stigma. A report reviewing substance use treatment in Indiana as per urban/rural divides found that rural and rural/mixed counties were less likely to have access to substance use treatment facilities and faced additional barriers such as lack of access to specialized care, inferior quality of care, having to pay more for services, and stigma [33]. Our data suggest that policymakers at the state-level should seek to expand the availability of substance use prevention and treatment services in underserved rural Indiana counties to fill this gap. Additionally, along with medical treatment facilities, emphasis should be placed on education-based programs and services for parents and other youth-involved populations to reduce stigma and provide up-to-date information on current substance use trends. Consistent with our findings, creative modes of delivery, including asynchronous online materials (perhaps that could also be downloaded and printed for those without internet access) may be particularly useful in these populations. In Indiana, leveraging Extension, which can help to maintain community support within local contexts, is a promising mode of delivery for up-to-date information.

## Conclusions

### Barriers and limitations

The findings from this study should be interpreted in the context of its limitations. First, as this was a pilot study, programs were conducted in only 8 of 92 Indiana counties, and each program was attended by a small number of participants. Therefore, our findings cannot be generalized to the broader population without implementing the program on a larger scale. In addition, without targeted recruitment strategies, the participants in our qualitative focus group discussions were likely to be more engaged and invested in adolescent substance use prevention than the general population. In fact, one of the main barriers that we faced during the implementation of this program was the limited participation from parents in the general community. As mentioned in the Results section, a majority of the program attendees were youth-involved professionals who discussed the difficulties they themselves had often faced in engaging parents in community programming efforts. This is a commonly faced implementation barrier, but also one that has been reported specifically in Indiana with regard to substance use programming for parents in particular [38]. The lack of participation from parents could be due to structural barriers that hamper their ability to access such resources—for instance, limited transportation or childcare services, or families’ struggles with much more basic needs such as food, that may prevent them from budgeting for extra resources. One solution to this barrier, as recommended by several of our program attendees, could be to incentivize the program by offering meals during the programming, assist with transportation to and from the program, or offer childcare services for the duration of the program. Finally, although focus group discussions allow for an in-depth look at the narratives of program participants, we cannot make conclusions regarding the effectiveness of our program without additional evaluation data. Instead, lessons learned during the design and implementation of this program, as well as our qualitative program assessment findings, can inform future adolescent substance use prevention efforts at the community level.

### Strengths and future directions

Despite its limitations, this study had several strengths and contributes to the broader adolescent substance use prevention literature by addressing the design, implementation, and assessment of an evidence-informed, community-based program that can easily be disseminated state-wide. Prevention efforts implemented by state agencies play an important role in influencing population-level health outcomes and tackling barriers to public health impact. Particularly, state-level initiatives ensure that evidence-based programs are adequately funded, well-implemented, and sustained, and take into account valuable input from prevention researchers and practitioners [39]. In Indiana, state-level policies are also critical in expanding the availability of services in rural and underserved counties [31]. Additionally, CBPR-based approaches to program development allow for the equitable involvement of community members in defining their own health-related needs, identifying which audiences to target, and offering unique perspectives on how to collect data and disseminate findings [19].

With these policy implications in mind, our pilot study included a collaboration with Purdue Extension—a well-established state-level community stakeholder—and key elements of CBPR in the creation and dissemination of a prevention program that addresses one of Indiana’s top public health concerns. Our findings offer crucial lessons learned during community-based program implementation and follow-up qualitative data collection.

 Attendees across all eight counties expressed their appreciation for the program, and we designed it to be applicable to a wide range of audiences. As it was disseminated in some of Indiana’s most “at-risk” counties for adolescent substance use [40], this pilot study provides insight into the concerns, existing resources, and needs of some of the state’s more underserved populations. Finally, the program content was entirely evidence-informed, using up-to-date statistics and findings to provide parents and other youth-involved adults with real-life strategies to prevent substance use in teens. This content is also easily deliverable in an online format with handouts and resources, therefore making it translatable across a wide range of contexts.

Since the pilot program, Purdue Extension has elected to co-brand and continue to offer the program across the state (https://www.purdue.edu/hhs/extension/program7/). In response to the feedback from the pilot program and through the co-branding, this program has transitioned to be an online short-course (1.5-2 h, that can be given in one or two settings), with updated rates of use, slag terms, and resources list, and enhanced online resources and handouts. This improved program will be evaluated by the Purdue Extension program evaluation survey that is filled out after each Extension program offering.

## Data Availability

Excerpts of the transcripts used for data analysis that are relevant to the study are available from the corresponding authors on reasonable request.

## References

[1] Irons DE, Iacono WG, McGue M. Tests of the effects of adolescent early alcohol exposures on adult outcomes. Addiction [Internet]. 2015 Feb;110(2):269–78. Available from: http://doi.wiley.com/10.1111/add.12747. 10.1111/add.12747 PMC4459504 25251778

[2] Johnston LD, Malley PMO, Bachman JG, Schulenberg JE. Monitoring the Future, Key Findings on Adolescent Drug Use. 2021;

[3] Jun M, Gassman R, Agley JD, King R, Samuel S, Lee J (2020). Indiana Youth Survey-2020.

[4] Gassman R, Jun M, Samuel S, Agley JD, Lee J, Wolf J. Indiana Youth Survey - 2018. 2018.

[5] U.S. Department of Health & Human Services (HHS) Office of the Surgeon General. Facing addiction in America: The surgeon general’s report on alcohol, drugs, and health. Vol. 49. Washington D.C.; 2016. 28252892

[6] State of Indiana. Commission on Improving the Status of Children in Indiana: Strategic Plan 2020-2022. 2020.

[7] State of Indiana. Commission on Improving the Status of Children in Indiana: Strategic Plan 2017-2019. 2019.

[8] Gray KM, Squeglia LM (2018). Research Review: What have we learned about adolescent substance use?. J Child Psychol Psychiatry Allied Discip.

[9] Kessler RC, Berglund P, Demler O, Jin R, Merikangas KR, Walters EE. Lifetime Prevalence and Age-of-Onset Distributions of. Arch Gen Psychiatry [Internet]. 2005;62(June):593–602. Available from: http://archpsyc.jamanetwork.com/article.aspx?doi=10.1001/archpsyc.62.6.593. 10.1001/archpsyc.62.6.593 15939837

[10] Compton WM, Thomas YF, Stinson FS, Grant BF. Prevalence, Correlates, Disability, and Comorbidity of DSM-IV Drug Abuse and Dependence in the United States. Arch Gen Psychiatry [Internet]. 2007 May 1;64(5):566. Available from: http://archpsyc.jamanetwork.com/article.aspx?doi=10.1001/archpsyc.64.5.566. 10.1001/archpsyc.64.5.566 17485608

[11] National Institute on Drug Abuse. NIDA. Prevention Principles [Internet]. 2020 [cited 2021 Mar 24]. Available from: https://www.drugabuse.gov/publications/preventing-drug-use-among-children-adolescents/prevention-principles.

[12] Marel C, Sunderland M, Mills KL, Slade T, Teesson M, Chapman C. Conditional probabilities of substance use disorders and associated risk factors: Progression from first use to use disorder on alcohol, cannabis, stimulants, sedatives and opioids. Drug Alcohol Depend [Internet]. 2019;194(September 2018):136–42. Available from: 10.1016/j.drugalcdep.2018.10.010. 10.1016/j.drugalcdep.2018.10.010 30439610

[13] Hodder RK, Freund M, Wolfenden L, Bowman J, Nepal S, Dray J, et al. Systematic review of universal school-based ‘resilience’ interventions targeting adolescent tobacco, alcohol or illicit substance use: A meta-analysis. Prev Med (Baltim) [Internet]. 2017;100:248–68. Available from: 10.1016/j.ypmed.2017.04.003. 10.1016/j.ypmed.2017.04.003 28390835

[14] Faggiano F, Minozzi S, Versino E, Buscemi D. Universal school-based prevention for illicit drug use. Cochrane Database Syst Rev [Internet]. 2014 Dec 1; Available from: https://doi.wiley.com/10.1002/14651858.CD003020.pub3. 10.1002/14651858.CD003020.pub3 PMC6483627 25435250

[15] Foxcroft DR, Tsertsvadze A. Universal school-based prevention programs for alcohol misuse in young people. Cochrane Database Syst Rev [Internet]. 2011 May 11; Available from: https://doi.wiley.com/10.1002/14651858.CD009113. 10.1002/14651858.CD009113 21563171

[16] Kuntsche S, Kuntsche E. Parent-based interventions for preventing or reducing adolescent substance use - A systematic literature review. Clin Psychol Rev [Internet]. 2016;45:89–101. Available from: 10.1016/j.cpr.2016.02.004. 10.1016/j.cpr.2016.02.004 27111301

[17] Holcomb E, McLelland J. A Strategic Approach to Addressing Substance Abuse in Indiana. 2017.

[18] Green LW, Royal Society of Canada, BC Consortium for Health Promotion Research. Study of participatory research in health promotion: Review and recommendations for the development of participatory research in health promotion in Canada. 1995.

[19] Minkler M, Wallerstein N, editors. Community-based participatory research for health: From process to outcomes. John Wiley & Sons; 2011.

[20] Wallerstein N, Duran B, Oetzel JG, Minkler M, editors. Community-based participatory research for health: Advancing social and health equity. John Wiley & Sons; 2017.

[21] Collins SE, Clifasefi SL, Stanton J, The LEAP Advisory Board, Straits KJE, Gil-Kashiwabara E, et al. Community-based participatory research (CBPR): Towards equitable involvement of community in psychology research. Am Psychol [Internet]. 2018 Oct;73(7):884–98. Available from: http://doi.apa.org/getdoi.cfm?doi=10.1037/amp0000167. 10.1037/amp0000167 PMC6054913 29355352

[22] Casey MA. Focus groups: A practical guide for applied research. Sage Publications; 2009.

[23] Knowles M. Focus Groups: A tool for program development and evaluation. 2015.

[24] Adler K, Salanterä S, Zumstein-Shaha M (2019). Focus Group Interviews in Child, Youth, and Parent Research: An Integrative Literature Review. Int J Qual Methods.

[25] Wutich A, Lant T, White DD, Larson KL, Gartin M. Comparing Focus Group and Individual Responses on Sensitive Topics: A Study of Water Decision Makers in a Desert City. Field methods [Internet]. 2010 Feb;22(1):88–110. Available from: http://journals.sagepub.com/doi/10.1177/1525822X09349918.

[26] Kidd PS, Parshall MB. Getting the Focus and the Group: Enhancing Analytical Rigor in Focus Group Research. Qual Health Res [Internet]. 2000 May 1;10(3):293–308. Available from: http://journals.sagepub.com/doi/10.1177/104973200129118453. 10.1177/104973200129118453 10947477

[27] Saldaña J. The coding manual for qualitative researchers. Sage; 2021.

[28] Guest G, MacQueen K., Namey EE. Validity and Reliability (Credibility and Dependability) in Qualitative Research and Data Analysis. In: Applied Thematic Analysis [Internet]. 2455 Teller Road, Thousand Oaks California 91320 United States: SAGE Publications, Inc.; 2012. p. 79–106. Available from: http://methods.sagepub.com/book/applied-thematic-analysis/n4.xml.

[29] King N, Brooks J. Thematic Analysis in Organisational Research. SAGE Handb Qual Bus Manag Res Methods Methods Challenges. 2018;219–36.

[30] Ritchie J, Spencer L, Bryman A, Burgess RG. Analysing qualitative data. 1994.

[31] Nowell LS, Norris JM, White DE, Moules NJ (2017). Thematic Analysis: Striving to Meet the Trustworthiness Criteria. Int J Qual Methods.

[32] Carter N, Bryant-Lukosius D, Dicenso A, Blythe J, Neville AJ (2014). The use of triangulation in qualitative research. Oncol Nurs Forum.

[33] IUPUI Center for Health Policy. Substance Abuse in Indiana: An Urban-Rural Perspective [Internet]. 2017. Available from: https://fsph.iupui.edu/doc/research-centers/SubstanceAbuseinIndiana-AnUrban-RuralPerspective.pdf.

[34] Yap MBH, Cheong TWK, Zaravinos-Tsakos F, Lubman DI, Jorm AF. Modifiable parenting factors associated with adolescent alcohol misuse: a systematic review and meta-analysis of longitudinal studies. Addiction [Internet]. 2017 Jul;112(7):1142–62. Available from: https://onlinelibrary.wiley.com/doi/10.1111/add.13785. 10.1111/add.13785 28178373

[35] Stockings E, Hall WD, Lynskey M, Morley KI, Reavley N, Strang J, et al. Prevention, early intervention, harm reduction, and treatment of substance use in young people. The Lancet Psychiatry [Internet]. 2016 Mar;3(3):280–96. Available from: https://linkinghub.elsevier.com/retrieve/pii/S221503661600002X. 10.1016/S2215-0366(16)00002-X 26905481

[36] IUPUI Center for Health Policy. Community Conditions Favorable for Substance Use. 2018.

[37] Stagman S, Schwarz SW, Powers D. Adolescent Substance Use in the U.S.: Facts for Policymakers [Internet]. 2011. Available from: http://www.nccp.org/publications/pdf/text_1008.pdf.

[38] Indiana University Richard M. Fairbanks School of Public Health at IUPUI, Department of Health Policy & Management. Indiana Partnerships For Success (PFS) Final Report. 2020.

[39] Rhoades BL, Bumbarger BK, Moore JE. The role of a state-level prevention support system in promoting high-quality implementation and sustainability of evidence-based programs. Am J Community Psychol [Internet]. 2012 Dec;50(3–4):386–401. Available from: https://doi.wiley.com/10.1007/s10464-012-9502-1. 10.1007/s10464-012-9502-1 22441729

[40] IUPUI Center for Health Policy. Drug Use in Indiana: A Regional Perspective. 2019; 19.

